# Supplementary material for: Growing inequities by immigration group among older adults: population-based analysis of access to primary care and return to in-person visits during the COVID-19 pandemic in British Columbia, Canada
Source: BMC Prim Care. 2024 Sep 6;25:332. doi: 10.1186/s12875-024-02530-1 (PMC11378608; doi:10.1186/s12875-024-02530-1)
Supplement: Supplementary file 1 — Supplementary Material 1. [file 12875_2024_2530_MOESM1_ESM.docx]

**Appendix 1**. Full regression results

|  | **Age 20-39** | |  | **Age 40-59** | |  | **Age 60+** | |
| --- | --- | --- | --- | --- | --- | --- | --- | --- |
|  | **Unadjusted OR (95% CI)** | **Adjusted**  **OR (95% CI)** |  | **Unadjusted OR (95% CI)** | **Adjusted**  **OR (95% CI)** |  | **Unadjusted OR (95% CI)** | **Adjusted**  **OR (95% CI)** |
| **Immigration group (reference is non-immigrant)** | | | | | | | | |
| Long-term immigrant (5+ years) | 0.89 (0.89, 0.90) | 0.86 (0.85, 0.87) |  | 0.92 (0.91, 0.93) | 0.92 (0.91, 0.93) |  | 0.52 (0.51, 0.52) | 0.56 (0.55, 0.57) |
| Recent immigrant (<5 years) | 1.10 (1.08, 1.11) | 1.05 (1.03, 1.06) |  | 0.81 (0.79, 0.82) | 0.87 (0.84, 0.89) |  | 0.53 (0.51, 0.56) | 0.58 (0.53, 0.62) |
| Recent immigrant (<5 years, low English) | 1.28 (1.23, 1.33) | 1.19 (1.13, 1.26) |  | 0.80 (0.77, 0.83) | 0.80 (0.76, 0.85) |  | 0.38 (0.36, 0.39) | 0.42 (0.40, 0.45) |
| **Year (reference is 2019)** | | | | | | | | |
| 2020 | 0.77 (0.77, 0.77) | 0.77 (0.76, 0.77) |  | 0.77 (0.77, 0.78) | 0.78 (0.78, 0.79) |  | 0.77 (0.77, 0.78) | 0.75 (0.75, 0.76) |
| 2021 | 0.82 (0.81, 0.82) | 0.80 (0.80, 0.81) |  | 0.87 (0.86, 0.87) | 0.87 (0.86, 0.88) |  | 0.83 (0.82, 0.83) | 0.79 (0.78, 0.79) |
| **Interaction (immigration group and year, reference is 2019, non immigrant))** | | | | | | | | |
| Long-term immigrant (5+ years), 2020 |  | 0.91 (0.90, 0.92) |  |  | 0.89 (0.88, 0.90) |  |  | 0.95 (0.93, 0.96) |
| Recent immigrant (<5 years), 2020 |  | 0.89 (0.88, 0.91) |  |  | 0.95 (0.93, 0.98) |  |  | 0.85 (0.78, 0.92) |
| Recent immigrant (<5 years, low English), 2020 |  | 0.87 (0.82, 0.93) |  |  | 0.83 (0.78, 0.88) |  |  | 0.73 (0.68, 0.77) |
| Long-term immigrant (5+ years), 2021 |  | 0.94 (0.93, 0.95) |  |  | 0.95 (0.94, 0.96) |  |  | 1.00 (0.98, 1.01) |
| Recent immigrant (<5 years), 2021 |  | 0.91 (0.89, 0.93) |  |  | 0.95 (0.92, 0.98) |  |  | 0.96 (0.89, 1.04) |
| Recent immigrant (<5 years, low English), 2021 |  | 0.87 (0.82, 0.93) |  |  | 0.81 (0.76, 0.87) |  |  | 0.77 (0.73, 0.82) |
| **Age** | | | | | | | | |
| **25-29** | 1.05 (1.04, 1.06) | 1.07 (1.06, 1.08) | **45-49** | 1.06 (1.05, 1.07) | 1.01 (1.00, 1.02) | **65-69** | 1.26 (1.25, 1.27) | 1.17 (1.16, 1.18) |
| **30-34** | 1.19 (1.18, 1.20) | 1.22 (1.21, 1.23) | **50-54** | 1.20 (1.19, 1.21) | 1.08 (1.07, 1.09) | **70-74** | 1.59 (1.57, 1.61) | 1.34 (1.32, 1.36) |
| **35-39** | 1.29 (1.28, 1.30) | 1.33 (1.31, 1.34) | **55-59** | 1.36 (1.35, 1.37) | 1.17 (1.16, 1.18) | **75-79** | 1.91 (1.88, 1.93) | 1.42 (1.40, 1.44) |
| **20-24** | ref | ref | **40-44** | ref | ref | **80-84** | 1.66 (1.64, 1.69) | 1.18 (1.16, 1.21) |
|  |  |  |  |  |  | **85+** | 0.87 (0.85, 0.88) | 0.46 (0.45, 0.47) |
|  |  |  |  |  |  | **60-64** | ref | ref |
| **Sex/gender (reference is Male)** | | | | | | | | |
| F | 2.88 (2.86, 2.90) | 2.85 (2.83, 2.87) |  | 2.01 (1.99, 2.02) | 2.02 (2.00, 2.03) |  | 1.30 (1.29, 1.31) | 1.42 (1.41, 1.43) |
| **Rurality, (reference is metropolitan)** | | | | | | | | |
| Small urban | 0.91 (0.90, 0.92) | 0.89 (0.88, 0.90) |  | 1.00 (0.99, 1.00) | 0.97 (0.97, 0.98) |  | 1.33 (1.32, 1.35) | 1.16 (1.14, 1.17) |
| Rural/remote | 0.77 (0.77, 0.78) | 0.75 (0.74, 0.75) |  | 0.84 (0.83, 0.85) | 0.83 (0.82, 0.84) |  | 1.00 (0.99, 1.02) | 0.94 (0.93, 0.95) |
| Missing | 0.29 (0.28, 0.30) | 0.64 (0.50, 0.81) |  | 0.16 (0.16, 0.17) | 0.47 (0.38, 0.59) |  | 0.14 (0.13, 0.14) | 0.59 (0.48, 0.73) |
| **Neighbourhood income quintile (reference is highest)** |  |  |  |  |  |  |  |  |
| Lowest | 0.89 (0.88, 0.90) | 0.87 (0.86, 0.88) |  | 0.81 (0.81, 0.82) | 0.77 (0.76, 0.77) |  | 0.70 (0.69, 0.71) | 0.66 (0.65, 0.67) |
| 2nd | 0.98 (0.97, 0.99) | 0.96 (0.95, 0.97) |  | 0.93 (0.92, 0.94) | 0.89 (0.89, 0.90) |  | 0.84 (0.83, 0.86) | 0.81 (0.80, 0.82) |
| Middle | 1.01 (1.00, 1.02) | 0.98 (0.97, 0.99) |  | 0.95 (0.94, 0.96) | 0.92 (0.91, 0.93) |  | 0.89 (0.87, 0.90) | 0.86 (0.84, 0.87) |
| 4th | 1.02 (1.01, 1.03) | 1.00 (0.99, 1.01) |  | 0.97 (0.96, 0.98) | 0.96 (0.95, 0.97) |  | 0.92 (0.91, 0.94) | 0.90 (0.89, 0.92) |
| Missing | 0.29 (0.29, 0.30) | 0.43 (0.34, 0.55) |  | 0.16 (0.15, 0.16) | 0.36 (0.29, 0.45) |  | 0.11 (0.11, 0.12) | 0.22 (0.18, 0.27) |
| **Charlson Index** | | | | | | | | |
| ch1: Acute Myocardial Infarction | 2.27 (1.97, 2.61) | 2.57 (2.23, 2.96) |  | 3.13 (2.94, 3.33) | 3.45 (3.25, 3.67) |  | 3.41 (3.22, 3.61) | 3.61 (3.40, 3.83) |
| ch2: Congestive Heart Failure | 2.58 (2.39, 2.77) | 2.83 (2.62, 3.05) |  | 3.01 (2.86, 3.16) | 3.26 (3.10, 3.42) |  | 3.99 (3.85, 4.15) | 4.53 (4.36, 4.71) |
| ch3: Peripheral Vascular Disease | 3.20 (3.01, 3.40) | 3.20 (3.00, 3.42) |  | 3.37 (3.22, 3.53) | 3.48 (3.32, 3.64) |  | 4.79 (4.59, 5.01) | 4.95 (4.72, 5.18) |
| ch4: CerebroVascular Disease | 2.81 (2.67, 2.95) | 2.85 (2.71, 3.01) |  | 3.20 (3.09, 3.32) | 3.26 (3.14, 3.38) |  | 4.46 (4.32, 4.61) | 4.70 (4.54, 4.86) |
| ch5: Dementia | 2.71 (2.51, 2.93) | 3.08 (2.85, 3.33) |  | 3.14 (2.93, 3.35) | 3.21 (3.00, 3.44) |  | 3.71 (3.57, 3.85) | 4.36 (4.19, 4.54) |
| ch6: COPD / Other Resp Dis | 4.30 (4.23, 4.37) | 4.39 (4.32, 4.47) |  | 4.66 (4.59, 4.74) | 4.60 (4.52, 4.67) |  | 5.85 (5.72, 5.98) | 5.74 (5.62, 5.88) |
| ch7: Rheumatologic Dis | 3.90 (3.76, 4.04) | 3.34 (3.21, 3.48) |  | 4.23 (4.11, 4.35) | 3.74 (3.63, 3.85) |  | 5.41 (5.21, 5.62) | 5.03 (4.84, 5.24) |
| ch8: Digestive Ulcer | 3.45 (3.28, 3.63) | 3.63 (3.45, 3.83) |  | 3.28 (3.13, 3.43) | 3.30 (3.15, 3.46) |  | 4.22 (3.96, 4.50) | 4.37 (4.08, 4.67) |
| ch9: Mild Liver Dis | 3.45 (3.36, 3.55) | 3.75 (3.64, 3.85) |  | 3.43 (3.36, 3.51) | 3.57 (3.50, 3.66) |  | 3.87 (3.74, 4.01) | 4.16 (4.02, 4.32) |
| ch10: Diabetes | 4.76 (4.64, 4.89) | 4.74 (4.60, 4.87) |  | 6.66 (6.54, 6.79) | 7.12 (6.98, 7.25) |  | 6.84 (6.71, 6.96) | 7.53 (7.39, 7.67) |
| ch11: Diabetes w/ Chronic Comp | 3.89 (3.02, 5.00) | 3.93 (3.06, 5.04) |  | 4.09 (3.70, 4.52) | 4.35 (3.94, 4.79) |  | 4.82 (4.54, 5.12) | 4.93 (4.64, 5.25) |
| ch12: Hemi or Paraplegia | 4.00 (3.59, 4.46) | 4.34 (3.88, 4.86) |  | 3.30 (3.00, 3.64) | 3.47 (3.15, 3.83) |  | 2.78 (2.47, 3.13) | 2.72 (2.41, 3.08) |
| ch13: Renal Dis | 2.63 (2.48, 2.79) | 2.75 (2.59, 2.92) |  | 3.41 (3.27, 3.54) | 3.44 (3.30, 3.58) |  | 5.07 (4.93, 5.21) | 5.59 (5.43, 5.75) |
| ch14: Primary Cancer | 3.84 (3.76, 3.91) | 3.66 (3.59, 3.74) |  | 3.92 (3.85, 3.98) | 3.68 (3.61, 3.74) |  | 5.07 (4.97, 5.17) | 4.93 (4.83, 5.03) |
| ch15: Mod/Severe Liver Dis | 2.94 (2.74, 3.16) | 3.50 (3.26, 3.75) |  | 3.39 (3.14, 3.65) | 3.58 (3.33, 3.86) |  | 3.57 (3.18, 4.01) | 3.61 (3.20, 4.08) |
| ch16: Metastatic Cancer | 4.49 (4.28, 4.72) | 4.44 (4.21, 4.68) |  | 4.69 (4.46, 4.92) | 4.47 (4.26, 4.70) |  | 5.34 (5.03, 5.67) | 5.26 (4.94, 5.60) |
| ch17: HIV Infection | 2.89 (2.73, 3.06) | 2.70 (2.53, 2.88) |  | 2.32 (2.17, 2.49) | 2.90 (2.71, 3.12) |  | 1.70 (1.49, 1.93) | 1.98 (1.73, 2.27) |
